# Scoping Review on Search Queries and Social Media for Disease Surveillance: A Chronology of Innovation

**DOI:** 10.2196/jmir.2740

**Published:** 2013-07-18

**Authors:** Theresa Marie Bernardo, Andrijana Rajic, Ian Young, Katie Robiadek, Mai T Pham, Julie A Funk

**Affiliations:** ^1^College of Veterinary MedicineMichigan State UniversityEast Lansing, MIUnited States; ^2^Department of Population MedicineUniversity of GuelphGuelph, ONCanada; ^3^Laboratory for Foodborne ZoonosesPublic Health Agency of CanadaGuelph, ONCanada; ^4^Agriculture DepartmentFood and Agriculture OrganisationRomeItaly; ^5^Department of Political ScienceUniversity of Wisconsin-MadisonMadison, WIUnited States

**Keywords:** disease, surveillance, social media, review

## Abstract

**Background:**

The threat of a global pandemic posed by outbreaks of influenza H5N1 (1997) and Severe Acute Respiratory Syndrome (SARS, 2002), both diseases of zoonotic origin, provoked interest in improving early warning systems and reinforced the need for combining data from different sources. It led to the use of search query data from search engines such as Google and Yahoo! as an indicator of when and where influenza was occurring. This methodology has subsequently been extended to other diseases and has led to experimentation with new types of social media for disease surveillance.

**Objective:**

The objective of this scoping review was to formally assess the current state of knowledge regarding the use of search queries and social media for disease surveillance in order to inform future work on early detection and more effective mitigation of the effects of foodborne illness.

**Methods:**

Structured scoping review methods were used to identify, characterize, and evaluate all published primary research, expert review, and commentary articles regarding the use of social media in surveillance of infectious diseases from 2002-2011.

**Results:**

Thirty-two primary research articles and 19 reviews and case studies were identified as relevant. Most relevant citations were peer-reviewed journal articles (29/32, 91%) published in 2010-11 (28/32, 88%) and reported use of a Google program for surveillance of influenza. Only four primary research articles investigated social media in the context of foodborne disease or gastroenteritis. Most authors (21/32 articles, 66%) reported that social media-based surveillance had comparable performance when compared to an existing surveillance program. The most commonly reported strengths of social media surveillance programs included their effectiveness (21/32, 66%) and rapid detection of disease (21/32, 66%). The most commonly reported weaknesses were the potential for false positive (16/32, 50%) and false negative (11/32, 34%) results. Most authors (24/32, 75%) recommended that social media programs should primarily be used to support existing surveillance programs.

**Conclusions:**

The use of search queries and social media for disease surveillance are relatively recent phenomena (first reported in 2006). Both the tools themselves and the methodologies for exploiting them are evolving over time. While their accuracy, speed, and cost compare favorably with existing surveillance systems, the primary challenge is to refine the data signal by reducing surrounding noise. Further developments in digital disease surveillance have the potential to improve sensitivity and specificity, passively through advances in machine learning and actively through engagement of users. Adoption, even as supporting systems for existing surveillance, will entail a high level of familiarity with the tools and collaboration across jurisdictions.

## Introduction

Social media and search behavior produce vast new data sources of largely untapped scientific potential. The threat of a global pandemic posed by outbreaks of influenza H5N1 (1997) and Severe Acute Respiratory Syndrome (SARS, 2002), both diseases of zoonotic origin, provoked interest in improving early warning systems and reinforced the need for combining data from different sources. It led to novel ideas, for example, the use of search query data from search engines such as Google [1,2] and Yahoo! [3] as an indicator of when and where influenza was occurring. This methodology has subsequently been extended to other diseases and has led to experimentation with new types of social media for disease surveillance as they have become available. Despite the emergence of disease surveillance as an innovative use of social media and search engine technologies, there is limited knowledge regarding the scope and efficacy of this novel application. With the potential to greatly improve disease surveillance and mitigation, there is a significant need to understand key chronological developments of the tools and methodologies in order to inform future endeavors and to assess this technology application for potential end-users.

Traditional narrative literature reviews provide useful overviews of broad research fields; however, their utility to inform policy and decision making is limited due to the lack of methodological transparency in terms of study selection and possible bias in interpretation [4,5]. Scoping reviews are a structured and formal knowledge synthesis method that can be used to rapidly identify, characterize, and contextualize existing knowledge and gaps in research [6-8]. They represent a relatively new methodology that has increasingly been adopted in health and various other sectors [6], including recent applications in food safety and zoonotic public health [8-10]. The objective of this scoping review was to formally assess the current state of knowledge regarding the use of online search queries and social media for disease surveillance in order to inform and encourage future work on early detection and more effective mitigation of the effects of foodborne illness. We used structured scoping review methods to identify, characterize, and evaluate all published primary research, expert review, and commentary articles investigating or discussing the use of social media in surveillance of infectious diseases. The results are presented and discussed within the context of existing research knowledge, as well as the surveillance and policy needs, gaps, and opportunities.

## Methods

### Review Protocol and Team Expertise

The review was informed by an ongoing scoping review protocol that includes details of the review methodology, definitions, and all forms used in the project (see Multimedia Appendix 1). The review team consisted of all 6 co-authors with multidisciplinary expertise in epidemiology, infectious diseases, food safety and zoonoses, social media, and knowledge synthesis methods. An advisory committee consisting of 23 professionals from 12 government, academic, and civil society organizations and with expertise in epidemiology, food safety, risk communication, social media, spatial geography, computer science, and mathematics, was consulted throughout the review to ensure that relevant articles in their respective fields had not been missed. Preliminary results of the scoping review were presented to the advisory committee and stakeholder feedback was received at a related project initiative [11].

### Review Question and Scope

The review question was “What is the current state of knowledge about the use and efficacy of mining social media text and Web query trends for disease surveillance?” Social media were defined as a group of Internet-based online and mobile applications (eg, Twitter, Facebook) that allow the creation and exchange of user-generated content and data [12]. Disease surveillance was defined as the ongoing systematic collection and analysis of data and the provision of information that leads to action being taken to prevent and control a disease [13]. This included activities related to early detection, prevention, control, and eradication of sporadic cases and outbreaks, endemic and epidemic diseases, and infectious and chronic diseases. Threats were limited to biological (viruses, parasites, bacteria, and their toxins) and chemical agents (melamine, pesticides).

### Search Strategy

A pretested electronic search strategy was implemented in SciVerse Scopus (2002-2011) on August 16, 2011 (see Multimedia Appendix 1). The search strategy used a targeted combination of 17 social media and Internet-based tool terms (eg, blog, Internet), five disease terms (eg, outbreak), and five surveillance terms (eg, monitor). The search was limited to 2002 and onward to coincide with the wide use of Web 2.0 applications. A Scopus and Google Web search were also conducted to identify grey literature (eg, reports and newspaper articles); both were limited to the 100 most relevant hits. The Scopus Web search used the same search strategy as above, while the Google search used the query “social media for disease surveillance”. The reference lists of 11 topic-related articles were hand-searched to identify any additional relevant citations potentially missed by the initial search strategy.

### Scoping Review Management and Form Pretesting

All references were imported into the online bibliographic management program RefWorks and subsequently imported into DistillerSR, a Web-based systematic review software for relevance screening and data characterization and extraction.

Relevance screening and data characterization and extraction forms were pretested and refined to standardize interpretation among 4 reviewers before use. The relevance screening form was pretested on 20 abstracts by 5 reviewers (TB, AR, KR, MP, and JF), and reviewing proceeded when kappa agreements were >0.7. The data characterization and extraction form was also pretested on five articles by 3 reviewers (TB, KR, and JF). A high agreement and only minor editorial discrepancies were observed for a couple of open-ended questions. These were discussed among the team members and the most practical yet robust data characterization and extraction process was determined.

### Relevance Screening and Inclusion Criteria

Each abstract was screened for relevance against the inclusion and exclusion criteria by 2 independent reviewers (AR and JF, KR and TB). Any peer or non–peer-reviewed original research, review, or commentary article describing or discussing the use of social media in support of infectious disease surveillance (within the broad context of disease detection, prevention, and control) was considered relevant. Abstracts describing the use of social media within the context of educational or risk communication campaigns or strategies and those published in languages other than English, Spanish, or French were excluded due to their irrelevance to the scope of the review and limited resources for translation, respectively. Conflicts between reviewers were resolved by consensus or with the assistance of the corresponding author, when required. A list of all relevant articles identified at the relevance screening level was shared with the members of the Advisory Committee to identify if any potentially relevant citations were missed.

### Data Characterization and Extraction

The full papers of relevant abstracts were procured and subsequently assessed by one reviewer (KR) to confirm their relevance. To ensure the accuracy of the data characterization, a random subsample of 19 articles was also independently reviewed by a second reviewer: TB (n=10), JF (n=9), MP (n=5). At this stage, the data characterization and extraction were limited to articles investigating or discussing the review question within the context of infectious disease. An a priori developed data characterization and extraction form consisted of 20 closed (n=15 questions) and open (n=5) questions. The closed questions captured the article type and format, sector and targeted audience, definitions of social media (if reported), study/surveillance/jurisdiction objectives, type of social media and surveillance method description, investigation of comparison and/or accuracy of social media versus other surveillance systems, and reported strengths and challenges associated with social media–based surveillance. Conflicts between reviewers were resolved by consensus or with the assistance of the corresponding author, when required. Data extracted from primary research articles were downloaded as MS Excel spreadsheets, summarized, and charted using narrative synthesis, tables, and figures.

### Thematic Analysis

We conducted a thematic analysis of all identified review and case study articles (n=19) to determine the important characteristics, considerations, and challenges regarding the use of social media for infectious disease surveillance. Thematic analysis is a method of qualitative synthesis that involves the identification of key and recurrent themes and concepts from a body of literature [14]. The analysis was conducted by 2 independent reviewers (AR and IY) using an inductively developed form and code list (see Multimedia Appendix 1). The form and codes were informed by discussions from the workshop about the use of social media for disease surveillance and from reviewing a sample of five relevant articles. Both reviewers independently coded all documents and met periodically to compare and discuss their findings. After completion of coding, the 2 reviewers discussed and consolidated their results, then developed overall themes by grouping and consolidating codes that represented similar concepts.

## Results

### Search Strategy and Study Selection

**Figure 1:**
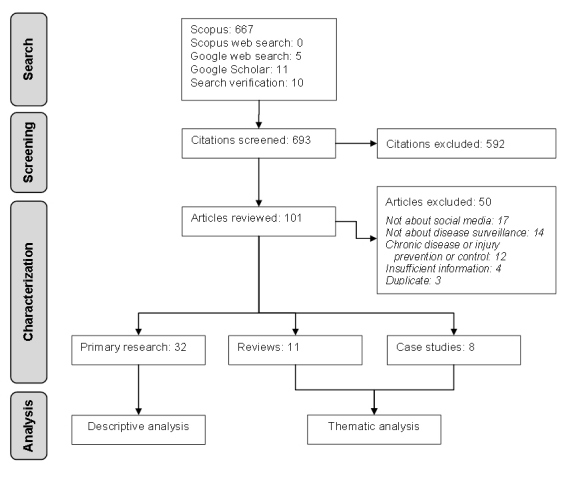
Scoping review flow chart.

The citation flow through various stages of the scoping review is shown in Figure 1. From 683 citations screened for relevance, 101 were considered potentially relevant and obtained as full articles.

### Data Characterization and Extraction

During data characterization and extraction, 32 primary research articles and 19 reviews and case studies were identified as relevant (Figure 1). The data characteristics of 32 relevant primary research articles are displayed in Table 1, and the full list of relevant articles from data characterization and extraction is available in Multimedia Appendix 1.

**Figure 2:**
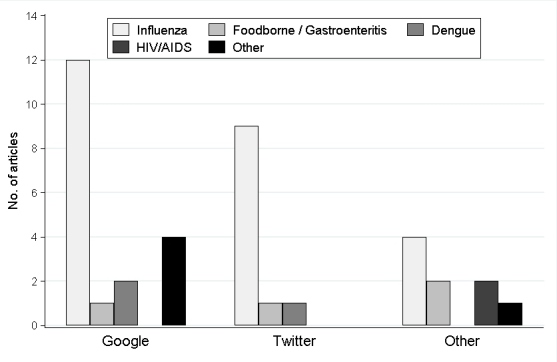
Distribution of published primary research investigating the use of social media programs for infectious disease surveillance from 2002-2011 (N=39 in this graph because some articles investigated more than one disease or used more than one social media program).

Most relevant citations were peer-reviewed journal articles (29/32, 91%) published in 2010 and 2011 (28/32, 88%) and reported the use of a Google program (17/32, 53%, eg, Google Trends, Flu Trends, or Insights for Search) for surveillance of influenza (23/32, 72%) (Table 1 and Figure 2). Only four primary research articles investigated social media in the context of foodborne disease or gastroenteritis (Table 1). None of the articles provided a definition for social media. However, two articles referred to the term “infodemiology”, which is defined as “the science of distribution and determinants of information in an electronic medium, specifically the Internet, or in a population, with the ultimate aim to inform public health and public policy” [1]. Use of infodemiology data for surveillance has been called “infoveillance” [1] or “digital disease detection” [15].

Most authors (21/32 articles, 66%) reported that the social media–based surveillance had good correlation when compared to an existing surveillance program (Table 2). The most commonly reported strengths of social media surveillance programs included their effectiveness (21/32, 66%) and rapid detection of disease trends (21/32, 66%). The most commonly reported weaknesses were the potential for false positive (16/32, 50%) and false negative (11/32, 34%) results (Table 2). Most authors (24/32, 75%) recommended that social media programs should primarily be used to support existing surveillance programs (Table 2).

**Table 1:** Characteristics of 32 primary research articles investigating the use of social media for infectious disease surveillance published from 2002-2011.

Question			No.	%
**Document type**				
	Peer-reviewed journal article		29	90.6
	Book chapter		1	3.1
	Workshop report		1	3.1
	Conference proceedings abstract		1	3.1
**Year of publication**				
	2011		13	40.6
	2010		15	46.9
	2006-2009		5	15.6
**Target audience** ^a^				
	Researchers and academics		29	90.6
	Practitioners, clinicians, or service providers		7	21.9
	Policy and decision makers		1	3.1
**Jurisdictional level of surveillance**				
	National		26	81.3
		USA	12	37.5
		Canada	2	6.3
		China	2	6.3
		UK	2	6.3
		Other^b^	8	25.0
	International		6	18.8
**Social media program investigated** ^a^				
	Google		17	53.1
		Google Trends	5	15.6
		Google Flu Trends	4	12.5
		Google Search	4	12.5
		Google Insights for Search	3	9.4
		Google AdSense	1	3.1
	Twitter		10	31.3
	Yahoo		2	6.3
		Yahoo Search	1	3.1
		Yahoo Knowledge public health forums	1	3.1
	Other search engine^c^		3	9.4
	Blogs or Web forum		2	6.3
**Infectious disease investigated** ^a^				
	Influenza (seasonal and highly pathogenic)		23	71.9
	Foodborne disease / gastroenteritis		4	12.5
	Dengue		3	9.4
	HIV/AIDS		2	6.3
	Other^d^		4	12.5

^a^Multiple answers allowed per article (ie, percentages do not add to 100%).

^b^Other countries included Australia, Brazil, France, Germany, Japan, Spain, Sweden, and Taiwan.

^c^Included Baidu (n=2) and Vardguiden (n=1).

^d^Other diseases included scarlet fever, tuberculosis, Lyme disease, methicillin-resistant Staphylococcus aureus, chickenpox, and ophthalmologic conditions.

**Table 2:** Characteristics of social media programs for infectious disease surveillance as reported in 32 primary research articles published from 2002-2011.

Question		No.	%
**Accuracy of the social media program compared to an existing surveillance program** ^a^			
	The compared systems showed good correlation	21	65.6
	The social media program was more accurate	2	6.3
	The existing program was more accurate	2	6.3
	Not reported	1	3.1
	No comparison conducted	7	21.9
**Reported strengths of social media programs for infectious disease surveillance** ^b^			
	Effective	21	65.6
	Faster response/detection	21	65.6
	Cost-effective	9	28.1
	Easy to access	7	21.9
	User-friendly	4	12.5
	Unique/global population as data source	4	12.5
	Less resource intensive	3	9.4
	Flexible	3	9.4
**Reported weaknesses of social media programs for infectious disease surveillance** ^b^			
	Potential for false positives (eg, increased searching due to media reporting)	16	50.0
	Potential for false negatives (eg, social media users might not represent general public)	11	34.4
	Variability in the function of different social media tools	4	12.5
	User information privacy concerns	2	6.3
	Sufficient skills and timely use needed to be effective	1	3.1
**Reported recommendations for the use of social media programs for infectious disease surveillance** ^b^			
	Should primarily support existing programs	24	75.0
	Should be used in the future when the methods are better validated and evaluated	3	9.4
	Should be used as a proxy for existing programs or when no traditional surveillance program exists	3	9.4
	Not reported	3	9.4

^a^One article had two responses based on differences in program performance for different diseases investigated.

^b^Multiple answers allowed per article (ie, percentages do not add to 100%).

### Thematic Analysis

**Figure 3:**
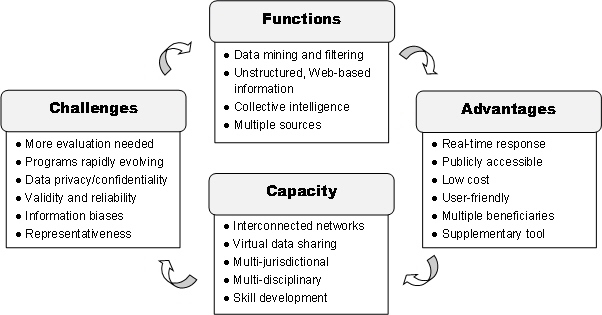
Key characteristics of social media programs for infectious disease surveillance.

Four thematic areas were identified as key characteristics of social media-based surveillance in the context of infectious disease (Figure 3). The first theme relates to the methodological aspects of the programs. In general, a variety of ontologies and search algorithms is used to synthesize and filter unstructured information from a variety of Web-based sources [15-21]. These sources can include news aggregates (eg, ProMed-mail), social media platforms (eg, Twitter), blogs, and search engine queries (eg, Google). Data sources can be characterized further as supply-based (eg, blogs and social media) or demand-based (eg, search behaviors) [18]. The overall principle behind these programs is that they aim to make sense of the public’s “collective intelligence” for purposes of early detection and effective control of infectious disease [15,18].

A second identified theme was the necessary capacity for developing these programs in practice. A multidisciplinary and multijurisdictional approach is needed to allow adequate data collection, exchange, and evaluation and communication across multiple jurisdictions and wide geographical areas [18,20,21]. Social media programs can allow international networks of food safety, public health, and other professionals to communicate via virtual networks, which can facilitate collaborations and support public health response infrastructure [19-22]. One example is virtual situation rooms using a three-dimensional interface, where public health professionals can collaborate and discuss surveillance data in real-time [20]. However, government and public health officials must be adequately trained and skilled in order to utilize these tools for disease surveillance in an effective and timely way [15,21].

Several advantages were frequently pointed out regarding the social media-based surveillance programs for infectious disease. First, the identification of disease trends in real-time, which can contribute to rapid outbreak detection and response [15,17-21]. In addition, they tend to be openly accessible to the public, be low cost or free, have a familiar and user-friendly interface, and have potential applications and benefits for multiple end-users (eg, public health officials, media, and travellers) [15,17-21,23,24]. In confirmation with our analysis of primary research articles (Table 2), these programs are primarily recommended as supplementary applications to existing surveillance programs, or as Madoff et al [25] note: “another tool in the surveillance toolbox.”

Finally, multiple challenges to the use of social media programs for infectious disease surveillance were identified. One of the most important challenges relates to the validity and reliability of the data analysis. For example, several authors discussed the need to properly filter out background noise (eg, people searching out of curiosity rather than illness) to ensure that the surveillance data reflect actual disease trends and are not a result of heightened media exposure or other biases [15,17,19,21,23,25,26]. In addition, there are still certain segments of the population that do not regularly use the Internet or social media programs, particularly in developing countries, so the users of these programs may not accurately represent the general population [20,24,25,27]. Another challenge relates to the ownership of the data and the issues surrounding user information privacy and confidentiality [21,27]. Most authors agreed that more evaluation, validation, and development of these programs is needed before they should be widely used in practice [17,20,25,26,28].

## Discussion

### Overview

The relevant research identified by this scoping review included a total of 51 articles, most of which were published since 2010 and investigated applications for enhancing influenza surveillance. This low to moderate yield of research activity was expected, as neither Web searches nor social media were developed with the objective of disease surveillance in mind and they are relatively recent phenomenon. As is frequently the case with innovation, new uses of existing tools are driven by necessity and/or opportunity.

Experimentation with search queries and social media for disease surveillance appears to reflect the chronological availability of new tools and the concurrent disease surveillance challenges, as well as the development of data mining and machine learning techniques. This may explain, in part, why the most common approach was to use Google-related search tools, as their chronological development preceded other social media tools, as well as Google’s more global scope in availability for application.

### Chronology of Development

As the use of Web searches to obtain health information became commonplace, researchers turned from following the number of people searching for health information, to looking at whether the frequency of searches on particular subjects harbored useful data, such as clues to disease outbreaks. The earliest article identified by this scoping review by Eysenbach [1] was published before search query data were widely available. Eysenbach devised a clever method to circumvent this restriction and acquired data on searches related to influenza through a strategic combination of bids for targeted Google keywords and placement of an influenza-related advertisement. He then developed a model for detecting influenza outbreaks in Canada based on changes in Canadians’ searches for information on influenza. When evaluated against the gold standard for influenza surveillance (reports by sentinel physicians of clinical encounters with influenza-like illness), the model proved to be more timely, accurate, and inexpensive [1]. The benefits reported in this earliest publication reflect the main benefits of social media-facilitated disease surveillance identified in the literature included in the scoping review.

The infectious disease most commonly evaluated using social media surveillance techniques was influenza, which is not surprising as these tools became available during a period of heightened sensitivity to the threat of a global pandemic following outbreaks of influenza H5N1 in 1997 and SARS in 2002. A study by Polgreen et al [3] found that the frequency of searches for influenza had predictive potential in the United States, looking at data over a longer time period (2004 to 2008) and using a different search engine (Yahoo!) than Eysenbach [1]. They were able to predict an increase in positive cultures for influenza 1-3 weeks before the increase occurred (*P*<.001) and an increase in mortality attributable to pneumonia and influenza up to 5 weeks in advance (*P*<.001). Two of the authors were employees at Yahoo!, which accounts for their access to search data [3].

In 2009, a letter authored by employees of Google and the Centers for Disease Control and Prevention (CDC) published in *Nature* described a large-scale effort to use Google search queries to track influenza [2]. A model was created based on the top 45 queries most correlated with CDC data on influenza-like illness. It consistently estimated the level of weekly influenza activity in each region of the United States with a 1-day reporting lag, which was 1-2 weeks ahead of reports by the CDC’s US Influenza Sentinel Provider Surveillance Network. Perhaps most importantly, results were made freely available online at Google Flu Trends website. This methodology was extended to Google Dengue Trends and was then generalized as Google Correlate, which allows users to enter their own search terms or time series data to find other terms that have a similar pattern of activity.

Pelat et al [29] demonstrated that using search queries for disease detection also functioned in another language (ie, French) and could be applied to other diseases (ie, gastroenteritis and chicken pox). The symptom of gastroenteritis, used as an indicator of foodborne illness, is of particular significance due to the difficulty in detecting foodborne illness in a timely manner. Whereas there is a lag of 1-2 weeks in tracking influenza, most foodborne disease outbreaks are not detected for several months after they occur, by which time the outbreak and opportunity for intervention are virtually over. A Chinese study by Zhou et al in 2010 [30] used both Baidu search queries and Baidu news articles to track infectious diseases including dysentery. They were able to reduce the distorting effect of disease-related news reports by using a combination of search frequency data and news count data. Surveillance reports from this effort were published 10-40 days ahead of the release of official reports from the Chinese government CDC.

Publications on the use of Twitter first appeared in 2010 and followed a similar pattern to the use of Internet search queries: they predominantly dealt with influenza (Figure 2) and ranged from content analysis of Twitter messages (tweets) related to the H1N1 outbreak [31,32] to demonstrating that tweets could accurately track an outbreak [31]. After analyzing over 570 million tweets, Culotta (2010) [33] concluded that “even extremely simple methods can result in quite accurate models” of influenza rates. Models are improved through judicious selection of keywords to track and by devising better methods to filter spurious tweets through natural language processing [34]. Content analysis of German tweets was also conducted for a number of diseases including influenza, norovirus, and salmonella [35].

### Geolocation

In addition to determining when an outbreak is occurring, it would be useful to know where it is occurring. Although geolocation was not targeted for evaluation in the scoping review, researchers included it as a possible use. The general physical location of a search query’s origin can often be identified from its associated Internet protocol (IP) address [2]. Although Twitter has an optional geolocation feature, a recent publication found that the prevalence of tweets with geolocation data was only 2%; however, city and state could be determined for 17% of user profiles using a simple text-matching approach [36]. Agreement between GPS data and text-matching was high (88%), as was the correlation between the number of geolocated tweets and state populations in the United States (ie, geolocated tweets were proportional to the state population) [36].

Two mapping systems were launched in 2006, BioCaster [16] and HealthMap [23], that monitor news feeds in multiple languages to provide real-time intelligence on emerging diseases around the world. Sources including news media, discussion sites such as ProMED-mail, and official reports of international organizations. HealthMap’s interface provides a means of organizing unstructured information based on geography, time, and infectious disease agent. HealthMap currently invites user input on missing outbreaks and includes a feature that solicits user contributions on influenza illness symptoms called “flu near you”.

The first articles describing mapping of tweets appeared in 2011. Signorini et al [31] created a Google map continuously updated with selected tweets to provide a real-time view of influenza-related public sentiment. Gomide et al [37] proposed a method for dengue surveillance in Brazil using four dimensions of Twitter data—volume, location, time, and content—in which they looked at the proportion of tweets expressing personal experience with dengue. Spatio-temporal analysis of dengue to detect clusters would enable government agencies to concentrate efforts in the right place at the right time.

### Participatory Surveillance

The potential of social media for epidemiology goes beyond the passive generation of new data streams from people, animals, food, or other sensors, and their movements. People can be actively involved in, or even instigate, epidemiological investigations. For example, postings in a Web forum about ill participants following a bike race in 2007 prompted the organizers to notify local public health authorities [38]. Messages and photos on the Web forum provided contextual clues as to the source (mud) of the outbreak (laboratory confirmed *Campylobacter jejuni*) that might have otherwise been missed, and an online questionnaire hastened the outbreak investigation [38].

Another example occurred in February 2011, when an Internet entrepreneur became sick after attending an international conference and posted a status update on Facebook [39]. Within a week, 80 other participants from around the world had self-identified and arrived at a potential diagnosis of legionellosis. The officer assigned to the case from the CDC joined the Facebook page to read the history of the outbreak and recommended appropriate diagnostic tests. This is an extreme example of participatory epidemiology whereby the investigation was initiated by those affected and epidemiologists were invited to participate. Social media is a breakthrough technology because it reduces the cost and difficulty of forming and working in groups, making it possible for loosely affiliated people to accomplish things that once were only possible through formal organizations [40].

### Potential for Adoption

Official reports by governments and international organizations were the primary source of disease intelligence during the 20th century. Unofficial reports were first taken into consideration by the moderated mailing list ProMED-mail, which was launched in 1994 [15]. Detection and investigation of “rumors” from news feeds and websites formed the basis of the Global Public Health Intelligence Network in 1997: a joint project of the Public Health Agency of Canada and the World Health Organization [1,15]. These examples set a precedent for the adoption of search queries and social media as a supplement to existing surveillance activities, in keeping with the reported recommendations of the scoping review (Table 2). Adoption, even as supporting systems for existing surveillance, will entail a high level of familiarity with the tools and collaboration across organizations and jurisdictions.

There is a growing body of evidence for the utility and accuracy of search queries in tracking diseases. The textual content of a tweet, however, differentiates it from search query data and may provide additional useful and timely information [31]. Computers can learn to distinguish useful messages based on word associations providing an automated method to deal with millions of tweets, using tools such as the Support Vector Machine (SVM)-based classifier [31,34]. The potential for false positives and false negatives was identified as one of the most commonly reported weaknesses by this scoping review (Table 2). One of the primary challenges is to refine the data signal by reducing surrounding noise. Further developments in digital disease surveillance have the potential to improve sensitivity and specificity: passively through advances in machine learning and actively through engagement of users.

Most of the identified research to date is associated with using Google search queries to detect seasonal or pandemic influenza days to weeks in advance of existing surveillance programs, but there are other promising areas for improvement. Just as influenza can be transported around the world in a matter of hours, our increasingly complex global food-supply chain presents a growing challenge to governments attempting to ensure a safe food supply in the face of dwindling budgets. Foodborne outbreaks can be notoriously difficult to detect as they can be widely distributed geographically and may be due to an ingredient that is found in a number of foods. Foodborne illness is also vastly underreported since most people who are affected do not seek medical attention nor receive laboratory confirmation of the causative agent (necessary steps to trigger declaration of an outbreak). Newkirk et al [41] make the case for using real-time data from social media to bypass significant delays in traditional foodborne surveillance activities, estimating a potential savings of 5-19 days in the reporting timeline for salmonellosis.

### Limitations

A potential limitation of this review is that only one electronic database was used to identify literature; however, we believe that our search verification strategy helped to limit this potential bias and are confident that the review was robust, results are accurate, and all relevant articles published during the study period were included. Another limitation of this review is the potential bias introduced by having only 1 reviewer extract data from the primary research articles during the data characterization and extraction step. However, we are confident that these results are accurate given that only minor conflicts were identified among the sample of articles verified by a second reviewer. In addition, many of our key results and conclusions correspond to and build upon those of other recently published reviews in this area [41,42].

### Conclusions

The use of search queries and social media for disease surveillance are relatively recent phenomena. Both the tools themselves and the methodologies for exploiting them are evolving over time. The growing evidence base regarding the utility of social media for disease surveillance will hopefully encourage academia, industry, the public service, and international organizations to consider social media in a serious light, particularly as a means of engagement rather than just disseminating information. While their accuracy, speed, and cost compare favorably with existing surveillance systems, the primary challenge is to refine the data signal by reducing surrounding noise. Further developments in digital disease surveillance have the potential to improve sensitivity and specificity: passively through advances in machine learning and actively through engagement of users. Although learning to use and adapt these new tools will take some time and effort, the greater challenge will be the multilevel collaboration among local, regional, national, and international authorities that will be required to use them most effectively.

## Multimedia Appendix 1

Supplementary material.

## Abbreviations

CDC: Centers for Disease Control and Prevention
SARS: Severe Acute Respiratory Syndrome
SVM: Support Vector Machine
