# Supplementary material for: Scoping Review on Search Queries and Social Media for Disease Surveillance: A Chronology of Innovation
Source: J Med Internet Res. 2013 Jul 18;15(7):e147. doi: 10.2196/jmir.2740 (PMC3785982; doi:10.2196/jmir.2740)
Supplement: Supplementary file 1 [file jmir_v15i7e147_app1.pdf]

Multimedia Appendix 1. Supplementary material.

Relevance screening form

Question	Options	Definitions/additional notes
RefID		
1.Does the abstract investigate the use of social media in disease surveillance?<br>*(Check all that apply)*<br><br>2.If "none of the above" was selected for question 1, does the abstract investigate a topic that may be interest to the project?<br>*(Check yes or no)*	1) Yes, general review or brief overview or commentary<br>2) Yes, within the context of primary research<br>3) None of the above<br><br>*If 3) "none of the above" is selected, abstract will be EXCLUDED*	*Yes, general review or overview or commentaries:*<br>• Includes general reviews or commentaries (from peer-reviewed articles to lay magazine or newspaper articles) describing, discussing and/or promoting the use of social media in support of disease surveillance (within the context of broad disease prevention and control defined below), with or without specific contextual examples.<br>*Yes, within the context of primary research*<br>• Any original research describing the use of social media in support of disease surveillance (within the context of broad disease prevention and control defined below), and reporting study (or program) design methods and results in replicable fashion of conventional, primary research.<br><br>*Social media are defined as a group of Internet-based online and mobile applications that allow the creation and exchange of* user-generated content *and data. (Examples*

		include Twitter, Facebook, Ushahidi, Google groups, data exhaust from Google searches, etc.).<br><br>*Disease surveillance is defined as the ongoing systematic collection and analysis of data and the provision of information which leads to action being taken to prevent and control a disease. Within the context of this study, disease prevention and control is defined broadly as any activity(ies) designed to protect public health (including human, animal and/or environmental health) from actual or potential health threats and their harmful consequences. This would include activities related to (early) detection of disease (including new disease, sporadic or outbreaks, endemic and epidemic, infectious and chronic), prevention, control and eradication. Threats are limited to biological and chemical. Articles describing the use of social media within the context of educational or risk communication campaigns or strategies will be excluded.*

Characterization form

Question	Options	Definitions/additional notes
RefID	Will be retrieved automatically from Distiller.	
Does the abstract investigate the <u>use of social media</u> in <u>disease surveillance</u>? *(Check all that apply)*	❒ Yes, general review or commentary.<br>❒ Yes, general review also describing case studies or detailed examples of social media use in support of disease prevention and control.<br>❒ Yes, primary research.<br>❒ None of the above, specify reason for exclusion: ____________________<br><br>*If "None of the above" is selected, article will be EXCLUDED*	*All definitions from RS form are applicable- CONFIRMATION OF RELEVANCE (full-article level).*<br><br>*Additional definitions:*<br>*Yes, general review or commentary:*<br>• Includes general reviews or commentaries of social media use in support of disease prevention and control, and only mentions examples without providing a detailed description.<br>*Yes, general review with case studies or examples:*<br>• Includes general reviews or commentaries as above but also illustrates the concepts through provision of detailed case-studies or examples.<br>*Yes, primary research:*<br>• The authors provide a detailed description of one or more social media tools within the context of prevention and

		control of one or more diseases or issues.
Within the context of this article, what is the main objective of social media in support of disease prevention and control?<br>*Check all that applies*	❒ Early warning, detection or control of disease outbreaks, epidemics or pandemics (due to infectious agents or toxic substances)<br>❒ Detection, control or management of chronic disease<br>❒ Other, specify: ___________________________	Articles describing the use of social media within the context of educational or risk communication campaigns or strategies will be excluded.<br><br>Note: Data extraction will initially be conducted for 1). Articles coded as 2) and 3) will proceed as time permits.
What type of document is the citation?	❒ Journal article<br>❒ Lay magazine article<br>❒ Newspaper article<br>❒ Government or research report<br>❒ Other, specify:____________	*Other*<br>• E.g. conference proceedings, thesis, book, web site, etc.
In what sector(s) is the article written?<br>*(Check all that apply)*	❒ Agriculture and agri-food, specify:_________________<br>❒ Health, specify disease:___________________<br>❒ Other, specify: __________________________	*Agriculture and agri-food*<br>• E.g., field crops, forestry, fishery, livestock<br>*Health*<br>• Specify the specific sub-field (e.g. public health, eHealth, infectious diseases, chronic diseases, mental health, foodborne disease) and specific disease, if applicable (e.g. dengue)<br><br>*Other: social sciences, business, education,*

		ecology, biology etc.
In the context of what population/target user group is the article written? *(Check all that apply)*	❒ Practitioners, clinicians, or service providers<br>❒ Policy- and/or decision-makers<br>❒ Researchers/academics<br>❒ Other, specify : _______________	*Practitioners, clinicians, or service providers*<br>• Article is targeted at clinical stakeholders (e.g. doctors), service providers or other practitioners<br>*Policy/decision makers*<br>• Article is targeted at public or government policy and/or decision makers<br>*Other*<br>• E.g. researchers/academics, general public, etc.
Is a definition of social media provided in this article?<br><br>What definitions are used to describe social media in this article? *(Check all that apply)*	❒ Provided by authors, specify: _______________________<br>❒ Other reference, specify:_________________<br>❒ No definition provided	*Provided by authors*<br>• Please type or copy and paste sentences used to define social media.<br>*Referenced*<br>• Please list the sentences used to define social media and the author/year of the *definition's reference source.*
What definitions of terms related to social media are provided in this article? *(Check all that apply)*	❒ Provided by authors, specify: ________________________<br>❒ Other reference, specify:_________________<br>❒ No definitions provided	*Provided by authors*<br>• Please list the *term(s)* or type or copy and paste sentences used to describe the social media.<br>*Referenced*

		• Please list the *term(s)* or sentences used to describe the social media and the author/year of the *definition's reference source.*
Within this article are there any references/citations that should be procured to obtain additional information about social media use in support of disease prevention and control?	[Textbox]	
What is the main study (review) objective as stated by the authors?	[Textbox]	Type or copy and paste as reported by authors.
Within the context of this article, what is the jurisdiction level of social media-based surveillance? *Check all that applies*	❐ International, specify: ___________________________<br>❐ Regional (greater than 1 country), specify: ___________<br>❐ National, specify: _________<br>❐ Other, specify:____________	
What specific social media tools are described or discussed by the authors? *Check all that applies*	❐ Google<br>❐ Twitter<br>❐ Other, please specify: ________________________	Examples of Google as a tool include data exhaust (data on searches conducted), or tools such as Correlate (not just simple Google searches conducted by the researchers).
Is there a description of the	❐ Yes<br>❐ No	Note: May not be applicable for review-

method as stated by the author(s)?	*If "Yes" is selected, then:*<br><br>What is the name of the social media tool?<br>____________________________________<br><br>What is the data source?<br>____________________________________<br><br>What is the data time period coverage?<br>____________________________________<br><br>What is the outcome measurement?<br>____________________________________<br><br>Is the model validated?<br>❒ Yes<br>❒ No<br>❒ Not applicable<br><br>Is there an additional social media tool described?<br>❒ Yes<br>❒ No<br><br>*If "Yes" is selected then:*<br><br>What is the name of the 2nd social media tool?<br>_______________________________<br><br>What is the data source for this 2nd social media tool?<br>_______________________________<br><br>What is the data time period coverage for this 2nd social media tool?<br>_______________________________<br><br>What is the outcome measurement for this 2nd social media tool?<br>_______________________________<br><br>Is the model validated?<br>❒ Yes<br>❒ No<br>❒ Not applicable	type papers<br><br>Type or copy and paste as reported by authors. Under 1), if description is not provided please write "not described".<br>If more than one social media tools or case studies or examples are reported within one article, please extract these data using separate form. Only questions for this section need to be completed more than once.

Do the authors compare (or correlate) the accuracy of social media-based surveillance concurrently with other surveillance systems?	❒ Not applicable<br>❒ Yes, it was compared to an existing sentinel or surveillance system, specify: ___________________________<br>❒ No, it was not compared to another surveillance system	Note: May not be applicable for review-type papers<br><br>Under 'specify': type or copy and paste as reported by authors. If description is not provided please write "not described".
Do the authors report that the social media-based surveillance is more accurate than other concurrently used surveillance systems?<br>*Check all that applies*	❒ Yes, social media-based surveillance is more accurate, specify:____________________<br>❒ No, but there is a good correlation between compared systems, specify: _________________________<br>❒ No, other surveillance system is more accurate, specify: _________________________<br>❒ Not reported, although the systems were concurrently compared.<br>❒ Not applicable, no concurrent comparison.	Note: May not be applicable for review-type papers<br><br>Type or copy and paste as reported by the authors.<br><br>Accuracy with respect to sensitivity (properly categorizing positives), specificity (properly categorizing negatives), and/or correctly predicting patterns in time and space
Do the authors report that the social media-based surveillance is effective in general within the context of disease prevention and control?	❒ Yes, please specify: ____________________<br>❒ Not reported	Type or copy and paste as reported by the authors.
Do the authors report that the social media-based surveillance is cost-effective within the context of disease prevention and control?	❒ Yes, please specify: ____________________<br>❒ Not reported	Type or copy and paste as reported by the authors.

Do the authors report the strengths of the social-media based surveillance? *Check all that applies*	❒ Ease of use<br>❒ Ease of access<br>❒ Time effective (less human labor)<br>❒ Faster<br>❒ Cost-effective<br>❒ Other, please specify:_______________<br>❒ No, not reported	
Do the authors report the weakness of the social-media based surveillance? *Check all that applies*	❒ Information overload<br>❒ Lack of validated methods (sensitivity and specificity)<br>❒ Other, please specify:_______________<br>❒ No, not reported	Type or copy and paste as reported by the authors.
What is overall recommendation by the authors regarding the social media-based surveillance within the context of disease prevention and control? *Check all that applies*	❒ Primarily in support of existing surveillance systems, please specify:______________<br>❒ Primarily in the context of future, when the methods are better validated or evaluated<br>❒ Other, please specify:<br>__________________________	Type or copy and paste as reported by the authors.
Do the authors report or comment on the human or professional-based social networks (outside of technology) within the context of disease prevention and control?	[Textbox]	Type or copy and paste as reported by the authors.
Do the authors report or comment on implications for the end user	[Textbox]	Type or copy and paste as reported by the authors.
Please provide a summary of any	[Textbox]	

additional important detail in the article that has not already been captured in this form. *(If applicable)*		

Thematic analysis form and code list

Question	Options	Definitions/additional notes
Does this article contain sufficient information for coding?	1) Yes<br>2) No, specify reason:___________<br><br>***If #2 is selected above, please submit the form without completing the remaining questions***	Reasons for exclusion could include short abstracts or articles with little to no relevant information about social media surveillance
Please indicate the key themes that are reviewed or described **in detail** in this review. *Check all that apply and indicate others as necessary.*	*Please see code list below for list of possible code options and their definitions*	Only include themes that are discussed in detail or depth in the article – and not those that are only briefly mentioned.
Please indicate 5-10 key messages from the article	___________________________	Please include a brief summary of 5-10 key messages from the article in point form.
Are there any other details or aspects of this article that should be noted? (E.g. key quotes)	___________________________	If there are any additional aspects or details about the article that should be noted, please enter those here.

Code List

Code	Definition
Accessibility	Data is publicly reported/available/accessible for free
Accuracy/validity/reliability	Discussion of sensitivity and/or specificity or validity/reliability of the programs (e.g. false alarms)
Beneficiaries	Discussion of who benefits from social media surveillance
Collaborative	Collaborations to integrate data across multiple jurisdictions/agencies/networks/countries
Confidentiality/privacy	Discussion of confidentiality of information or privacy as an issue
Costs/funding	Discussion of costs, cost-effectiveness of funding for programs
Data mining	Discussion of methods and processes to synthesize and extract necessary data
Data noise/volume	Discussion of need to filter data to reduce background "noise" and prevent information overload
Diverse end users	Discussion of different populations of end-users (e.g. researchers, practitioners, general public)
Evaluation/efficacy	Discussion of need to evaluate and verify the efficacy of the programs
Government role	Discussion of role of government agencies
Infrastructure/capacity	Discussion of infrastructure or capacity (e.g. skills, training) to implement social media surveillance

Informal/public data	Informal sources for data collection from general public information (e.g. blogs, web searches, RSS feeds); reflects public “collective intelligence”
International	Program is global or international in nature
Legal implications	Discussion of legal implications or responsibilities of social media surveillance (e.g. outbreak reporting obligations)
Mapping/visualization	Includes capabilities to map/visualize trends
Moderators	Discussion of need for moderators to manage information flow
Multiple data sources	Data is aggregated from multiple sources (e.g. blogs, searches, wikis, RSS feeds)
Networking/connections	Programs facilitate network building and connections among users
New technology tools	Use of mobile phones (e.g. iPhone), “texting”, Google, Twitter, Wikipedia, RSS feeds, Facebook, etc. for surveillance
Ownership	Discussion of who owns or is responsible for the data/program
Population/jurisdiction	Discussion of populations or jurisdictions where program is or can be implemented (e.g. developed vs. developing countries)
Detection of emerging diseases, outbreaks, pandemics	Programs can be used to detect emerging diseases, outbreaks, or pandemics
Rapid response/real-time	Importance of timely recognition and response of outbreaks/events
Supplementary role	Social media surveillance discussed in role supplementary or supportive of existing official/government programs
User-friendly	Discussion of need to ensure programs are easy to use/user friendly

List of search terms

The search strategy is a combination of key terms developed within the following domains: “social media/internet-based tools” AND “disease” AND “surveillance.”

Broad category	Search terms
1. Social media/internet-based tools	social media OR blog* OR microblog* OR crowdsourc* OR twitter OR facebook OR wiki* OR search engine OR (google OR internet OR web OR online) AND (social network OR social networking OR search OR query)
2. Disease	disease OR illness* OR outbreak OR epidemic OR pandemic
3. Surveillance	surveillance OR track* OR forecast* OR monitor* OR predict*

Limits: Starting in 2002

List of relevant articles from article characterization (n=51)

**Case study articles (n=8)**			
**Author**	**Title**	**Source**	**Year**
Brownstein JS, Freifeld CC, Reis BY and Mandl KD.	Surveillance Sans Frontieres: Internet-based emerging infectious disease intelligence and the HealthMap project	PLoS Med.	2008
Carneiro HA and Mylonakis E.	Google trends: A web-based tool for real-time surveillance of disease outbreaks	Clin. Infect. Dis.	2009
Collier N, Doan S, Kawazoe A, Goodwin RM, Conway M, Tateno Y, Ngo QH, Dien D, Kawtrakul A, Takeuchi K, Shigematsu M and Taniguchi K.	BioCaster: detecting public health rumors with a Web-based text mining system	Bioinformatics	2008
Eke PI.	Using social media for research and public health surveillance	J. Dent. Res.	2011
Eysenbach G.	Infodemiology and infoveillance: framework for an emerging set of public health informatics methods to analyze search, communication and publication behavior on the Internet.	J. Med. Internet Res.	2009
Kamel Boulos MN, Sanfilippo AP, Corley CD and Wheeler S.	Social Web mining and exploitation for serious applications: Technosocial Predictive Analytics and related technologies for public health, environmental and national security surveillance	Comput. Methods Programs Biomed.	2010
Madoff LC, Fisman DN and Kass-Hout T.	A new approach to monitoring dengue activity	PLoS Negl Trop. Dis.	2011
Schein R, Wilson K and Keelan J.	Literature Review on Effectiveness of the Use of Social Media: a report for Peel Public Health	*Report*	2010

**General review articles (n=11)**			
**Author**	**Title**	**Source**	**Year**
Anonymous	Google Flu Trends includes 14 European countries.	Euro Surveill.	2009
Brownstein JS, Freifeld CC and Madoff LC.	Digital disease detection - Harnessing the web for public health surveillance	New Engl. J.Med.	2009
Brownstein JS.	Using social media for disease surveillance	*CNN.com Blogs*	2011
Cook AR, Chen MIC and Lin RTP.	Internet search limitations and pandemic influenza, Singapore	Emerg. Infect. Dis.	2010
Dreesman J and Denecke K.	Challenges for signal generation from medical social media data	Stud. Health Technol.Inform.	2011
Greene M.	Constructing paths through social networks for disease surveillance	Proc SPIE Int Soc Opt Eng	2011
Greene M.	Epidemiological monitoring for emerging infectious diseases	Proc SPIE Int Soc Opt Eng	2010
Khan AS, Fleischauer A, Casani J and Groseclose SL.	The next public health revolution: Public health information fusion and social networks	Am. J. Public Health	2010
Lopes LF, Silva FAB, Couto F, Zamite J, Ferreira H, Sousa C and Silva MJ.	Epidemic marketplace: An information management system for epidemiological data	Lect. Notes Comput. Sci.	2010
Morozov E.	Swine flu: Twitter's power to misinform	Foreign Policy	2009
Strongin R.	Health reform in 140 characters	Med Device Diagn Ind	2010

Primary research articles (n=32)			
**Author**	**Title**	**Source**	**Year**
Achrekar H, Gandhe A, Lazarus R, Yu S- and Liu B.	Predicting flu trends using twitter data	IEEE Conf. Comput. Commun. Workshops	2011
Althouse BM, Ng YY and Cummings DA.	Prediction of dengue incidence using search query surveillance	PLoS Negl Trop.Dis.	2011
Aramaki E, Maskawa S and Morita M.	Twitter Catches the Flu: Detecting influenza epidemics using Twitter	Proceedings of the 2011 Conference on Empirical Methods in Natural Language Processing	2011;
Boyle JR, Sparks RS, Keijzers GB, Crilly JL, Lind JF and Ryan LM.	Prediction and surveillance of influenza epidemics	Med. J. Aust.	2011
Chan EH, Sahai V, Conrad C and Brownstein JS.	Using web search query data to monitor dengue epidemics: A new model for neglected tropical disease surveillance	PLoS. Negl. Trop. Dis.	2011
Chester TLS, Taylor M, Sandhu J, Forsting S, Ellis A, Stirling R and Galanis E.	Use of a Web Forum and an Online Questionnaire in the Detection and Investigation of an Outbreak	Online Journal of Public Health Informatics	2011
Chew C and Eysenbach G.	Pandemics in the age of Twitter: Content analysis of tweets during the 2009 H1N1 outbreak	PLoS ONE	2010
Corley CD, Cook DJ, Mikler AR and Singh KP.	Using Web and social media for influenza surveillance.	Adv. Exp. Med. Biol.	2010
Culotta A.	Detecting influenza outbreaks by analyzing Twitter messages		2010
de Quincey E and Kostkova P.	Early warning and outbreak detection using social networking websites: the potential of twitter	Electronic healthcare, Lecture Notes of the Institute for Computer Sciences, Social Informatics and Telecommunications Engineering	2010
Dukic VM, David MZ and Lauderdale DS.	Internet queries and methicillin-resistant staphylococcus aureus surveillance	Emerg. Infect. Dis.	2011
Dukic VM, Lopes LF and Polson NG.	Tracking flu epidemics using Google Flu Trends		2011
Eysenbach G.	Infodemiology: tracking flu-	AMIA Annu Symp	2006

	related searches on the web for syndromic surveillance.	Proc	
Ginsberg J, Mohebbi MH, Patel RS, Brammer L, Smolinski MS and Brilliant L.	Detecting influenza epidemics using search engine query data	Nature	2009
Goel S, Hofman JM, Lahaie S, Pennock DM and Watts DJ.	Predicting consumer behavior with web search	Proc. Natl. Acad. Sci. U.S.A.	2010
Gomide J, Veloso A, Meira W, Almeida V, Benevenuto F, Ferraz F and Teixeira M.	Dengue surveillance based on a computational model of spatio-temporal locality of Twitter	Proceedings of the ACM WebSci'11	2011
Hooten MB, Anderson J and Waller LA.	Assessing North American influenza dynamics with a statistical SIRS model	Spat.Spatio-temporal Epidemiol.	2010
Hulth A and Rydevik G.	GET WELL: An automated surveillance system for gaining new epidemiological knowledge	BMC Public Health	2011
Krieck M, Dreesman J, Otrusina L and Denecke K.	A New Age of Public Health: Identifying Disease Outbreaks by Analyzing Tweets	Health Web Science Workshop, ACM Web Science 2011 3rd International Conference on Web Science	2011
Ku Y, Chiu C, Zhang Y, Fan L and Chen H.	Global disease surveillance using social media: HIV/AIDS content intervention in web forums	ISI - IEEE Int. Conf. Intell. Secur. Informatics: Public Saf. Secur.	2010
Lampos V and Cristianini N.	Tracking the flu pandemic by monitoring the social web	Int. Workshop Cognitive Inf. Process.	2010
Lampos V, De Bie T and Cristianini N.	Flu detector-tracking epidemics on twitter	Lect. Notes Comput. Sci.	2010
Leffler CT, Davenport B and Chan D.	Frequency and seasonal variation of ophthalmology-related internet searches	Can. J. Ophthalmol.	2010
Luo Y, Zeng D, Cao Z, Zheng X, Wang Y, Wang Q and Zhao H.	Using multi-source web data for epidemic surveillance: A case study of the 2009 Influenza A (H1N1) pandemic in Beijing	Proc. IEEE Int. Conf. Serv. Oper. Logist., Informatics, SOLI	2010
Ortiz JR, Zhou H, Shay DK, Neuzil KM, Fowlkes AL and Goss CH.	Monitoring Influenza activity in the United States: A comparison of traditional surveillance systems with Google Flu Trends	PLoS ONE	2011

Pelat C, Turbelin C, Bar-Hen A, Flahault A and Valleron A-.	More diseases tracked by using google trends	Emerg. Infect. Dis.	2009
Polgreen PM, Chen Y, Pennock DM and Nelson FD.	Using internet searches for influenza surveillance	Clin. Infect. Dis.	2008
Seifter A, Schwarzwalder A, Geis K and Aucott J.	The utility of "Google Trends" for epidemiological research: Lyme disease as an example	Geospatial Health	2010
Signorini A, Segre AM and Polgreen PM.	The use of Twitter to track levels of disease activity and public concern in the U.S. during the influenza A H1N1 pandemic	PLoS ONE	2011
Valdivia Pérez A, Miguel Benito A, Escortell Mayor E and Monge Corella S.	Can the influenza epidemic be predicted by data from the Internet?	Gaceta Sanit.	2010
Valdivia A, López-Alcalde J, Vicente M, Pichiule M, Ruiz M and Ordobas M.	Monitoring influenza activity in Europe with Google Flu Trends: Comparison with the findings of sentinel physician networks - results for 2009-10	Eurosurveillance	2010
Zhou X- and Shen H-.	Notifiable infectious disease surveillance with data collected by search engine	J. Zhejiang Univ. Sci. C	2010
